# Absence of the *cbb*_3_ Terminal Oxidase Reveals an Active Oxygen-Dependent Cyclase Involved in Bacteriochlorophyll Biosynthesis in Rhodobacter sphaeroides

**DOI:** 10.1128/JB.00121-16

**Published:** 2016-07-13

**Authors:** Guangyu E. Chen, Daniel P. Canniffe, Elizabeth C. Martin, C. Neil Hunter

**Affiliations:** Department of Molecular Biology and Biotechnology, University of Sheffield, Sheffield, United Kingdom; Queen Mary, University of London

## Abstract

The characteristic green color associated with chlorophyll pigments results from the formation of an isocyclic fifth ring on the tetrapyrrole macrocycle during the biosynthesis of these important molecules. This reaction is catalyzed by two unrelated cyclase enzymes employing different chemistries. Oxygenic phototrophs such as plants and cyanobacteria utilize an oxygen-dependent enzyme, the major component of which is a diiron protein named AcsF, while BchE, an oxygen-sensitive [4Fe-4S] cluster protein, dominates in phototrophs inhabiting anoxic environments, such as the purple phototrophic bacterium Rhodobacter sphaeroides. We identify a potential *acsF* in this organism and assay for activity of the encoded protein in a strain lacking *bchE* under various aeration regimes. Initially, cells lacking *bchE* did not demonstrate AcsF activity under any condition tested. However, on removal of a gene encoding a subunit of the *cbb*_3_-type respiratory terminal oxidase, cells cultured under regimes ranging from oxic to micro-oxic exhibited cyclase activity, confirming the activity of the oxygen-dependent enzyme in this model organism. Potential reasons for the utilization of an oxygen-dependent enzyme in anoxygenic phototrophs are discussed.

**IMPORTANCE** The formation of the E ring of bacteriochlorophyll pigments is the least well characterized step in their biosynthesis, remaining enigmatic for over 60 years. Two unrelated enzymes catalyze this cyclization step; O_2_-dependent and O_2_-independent forms dominate in oxygenic and anoxygenic phototrophs, respectively. We uncover the activity of an O_2_-dependent enzyme in the anoxygenic purple phototrophic bacterium Rhodobacter sphaeroides, initially by inactivation of the high-affinity terminal respiratory oxidase, cytochrome *cbb*_3_. We propose that the O_2_-dependent form allows for the biosynthesis of a low level of bacteriochlorophyll under oxic conditions, so that a rapid initiation of photosynthetic processes is possible for this bacterium upon a reduction of oxygen tension.

## INTRODUCTION

**FIG 1:**
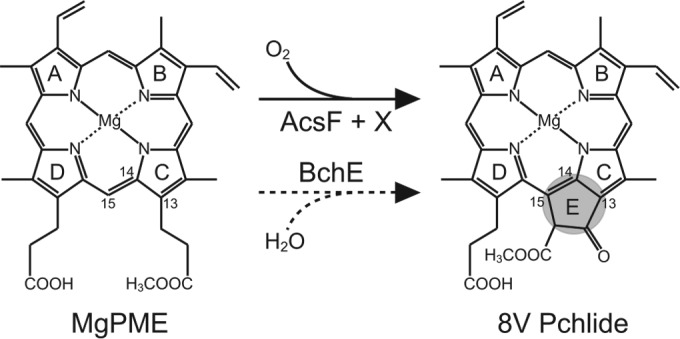
Isocyclic ring formation catalyzed by AcsF (solid arrow) and BchE (dashed arrow). IUPAC numbering of the relevant macrocycle carbons is indicated, and the catalyzed formation of ring E is highlighted. The oxygen sources for AcsF and BchE are molecular oxygen and water, respectively. X denotes the as-yet-unassigned subunit required for the oxidative reaction.

The (bacterio)chlorophylls [(B)Chls] are ubiquitous pigments employed by chlorophototrophic organisms for both light harvesting and photochemistry; thus, the elucidation of their biosynthetic pathways is of great importance. The least well characterized step in the common pathway for all of the (B)Chls is the formation of the isocyclic E ring, occurring via the oxidation and cyclization of the C-13 propionate group of magnesium protoporphyrin IX monomethyl ester (MgPME), producing 8-vinyl protochlorophyllide (8V Pchlide) (Fig. 1). The reaction is catalyzed by two distinct enzymes employing different chemistries: an oxygen-sensitive protein containing [4Fe-4S] and cobalamin prosthetic groups (1), which derives oxygen from water (2), and a diiron enzyme that requires molecular oxygen (3). Although an *in vitro* assay has not yet been described, the MgPME (oxygen-independent) cyclase enzyme is believed to be encoded by a single gene, *bchE* (4–6), which is essential for BChl biosynthesis in bacterial phototrophs inhabiting anoxic environments. The MgPME (oxygen-dependent) cyclase (EC 1.14.13.81) catalyzes this reaction in plants and cyanobacteria (7, 8) and has been demonstrated to require both soluble and membrane-bound components (9, 10). Interestingly, the first subunit assigned to the oxygen-dependent reaction was identified in the anoxygenic purple phototrophic bacterium Rubrivivax gelatinosus and was named AcsF (aerobic cyclization system Fe-containing subunit) (11); while the wild-type (WT) strain was able to synthesize BChl under oxic conditions, a mutant in *acsF* accumulated MgPME. It was subsequently discovered that Rbv. gelatinosus contained both forms of the cyclase, conferring the ability to synthesize BChl under different oxygen concentrations (12). Orthologs of *acsF* have since been studied in higher plants (13, 14) and cyanobacteria (15) as well as the green nonsulfur bacterium Chloroflexus aurantiacus (16). Recently, the distribution of *acsF* and *bchE* in the genomes of phototrophic proteobacteria has been investigated in detail (17); *acsF* is present in all of the aerobic anoxygenic phototrophs but is absent in the purple sulfur bacteria, while the majority of purple nonsulfur bacteria were found to contain both *acsF* and *bchE*.

Unlike the situation described for Rbv. gelatinosus, it has been well documented that BChl biosynthesis, and thus photosynthetic membrane assembly, is repressed by the presence of oxygen in Rhodobacter spp. of purple phototrophic bacteria (18). Under oxic conditions, it is possible to reduce cellular BChl contents to less than 1% of those of photosynthetic cells. As Rhodobacter sphaeroides transitions from oxic to micro-oxic conditions, this repression is lifted and the cell develops a system of pigmented membranes that house the photosynthetic apparatus (19–21). It was demonstrated that disruption of the Rba. sphaeroides *ccoNOQP* operon, previously shown to encode the *cbb*_3_-type terminal oxidase in Rhodobacter capsulatus (22), resulted in the development of this membrane system in the presence of O_2_ (23). Samuel Kaplan's laboratory studied various elements involved in the regulation of the maturation of this membrane in Rba. sphaeroides, showing that the rate of electron flow through the *cbb*_3_ oxidase and the redox state of the quinone pool in the photosynthetic membrane generate signals that regulate photosynthesis gene expression in this organism (24–28). An inhibitory signal generated by the *cbb*_3_ oxidase is transduced to the PrrBA two-component activation system, which controls the expression of most of the photosynthesis genes in response to O_2_, while the AppA/PpsR antirepressor/repressor system, modulated by TspO, monitors the redox state of the quinone pool. These systems, along with the assembly factors of the light-harvesting complexes (29), control the ultimate cellular levels and composition of the photosynthetic membrane.

**FIG 2:**
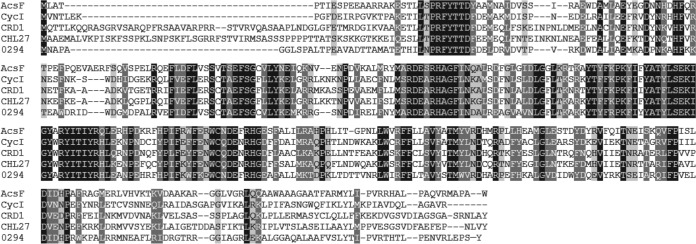
Amino acid sequence alignments of known AcsF proteins with RSP_0294. Sequences are those from Rbv. gelatinosus (AcsF), Synechocystis sp. PCC 6803 (CycI), Chlamydomonas reinhardtii (CRD1), and Arabidopsis thaliana (CHL27), aligned with RSP_0294 (0294). Conserved, highly similar, and similar residues are highlighted in black, dark gray, and light gray, respectively.

Aside from various antirepressor/repressor systems, the assembly of photosynthetic membranes will also be influenced by the characteristics of the biosynthetic enzymes involved, in terms of their tolerance to oxygen and/or their ability to use it as a substrate. A transition from oxic conditions to oxygen-limited growth initiates a developmental process that culminates in the assembly of the photosynthesis apparatus, and the early stages have to tolerate, and even use, the available oxygen. Thus, the presence of an oxygen-dependent cyclase could be beneficial, even though later stages of assembly rely on the oxygen-sensitive BchE cyclase. Thus, it is important to find out if there is an oxygen-dependent cyclase in Rba. sphaeroides. In this study, we identify an ortholog of *acsF* in Rba. sphaeroides, rsp_0294 (Fig. 2), which resides in the photosynthesis gene cluster (29). In order to test for activity of RSP_0294 as an oxygen-dependent cyclase component, we constructed a mutant lacking *bchE* in which we were initially unable to detect BChl *a*. Removal of the *cbb*_3_ oxidase in this background resulted in the accumulation of this pigment, confirming that rsp_0294 encodes an AcsF component of the cyclase enzyme. Subsequently, we discovered that in the presence or absence of this cytochrome, the Δ*bchE* mutant accumulates Zn-BChl *a*, potentially due to replacement of the central magnesium ion, as the pigment is not sequestered by the light-harvesting polypeptide apparatus. The reasons for the employment of an oxygen-dependent cyclase by an anoxygenic phototroph are discussed.

## MATERIALS AND METHODS

### Growth conditions.

Rba. sphaeroides strains were grown in the dark in a rotary shaker at 30°C in liquid M22+ medium (30) supplemented with 0.1% Casamino Acids. Differential aeration of cultures between oxic and micro-oxic was achieved by filling 250-ml Erlenmeyer flasks with 20 ml, 40 ml, 80 ml, and 160 ml of medium, with agitation at 150 rpm.

Escherichia coli strains JM109 (31) and S17-1 (32) transformed with pK18*mobsacB* plasmids were grown in a rotary shaker at 37°C in LB medium supplemented with 30 μg · ml^−1^ kanamycin. All strains and plasmids used in this study are listed in Table 1.

**TABLE 1:** List of strains and plasmids described in this study

Strain or plasmid	Description	Source or reference
E. coli		
JM109	Cloning strain for pK18*mobsacB* constructs	Promega
S17-1	Conjugation strain for pK18*mobsacB* constructs	32
Rba. sphaeroides		
2.4.1	WT strain	S. Kaplan^*a*^
Δ*bchE* mutant	Unmarked deletion mutant of *bchE* in WT	This study
Δ*ccoP* mutant	Unmarked deletion mutant of *ccoP* in WT	This study
Δ*bchE* Δ*ccoP* mutant	Unmarked deletion mutant of *ccoP* in Δ*bchE* strain	This study
Δ*bchE* Δ*ccoP* Δrsp_0294 mutant	Unmarked deletion mutant of rsp_0294 in Δ*bchE* Δ*ccoP* strain	This study
Plasmid pK18*mobsacB*	Allelic exchange vector; Km^r^	J. Armitage^*b*^

a Department of Microbiology and Molecular Genetics, The University of Texas Medical School, Houston, TX.

b Department of Biochemistry, University of Oxford, Oxford, United Kingdom.

### Construction of mutants of Rba. sphaeroides.

Rba. sphaeroides genes were deleted using the allelic exchange vector pK18*mobsacB* (33). Sequences up- and downstream of target genes were amplified with the relevant UpF and UpR primers and DownF and DownR primers, respectively. Sequences of all of the primers used in the present study can be found in Table S1 in the supplemental material. The up- and downstream PCR products were fused by overlap extension PCR, digested with the relevant restriction enzymes, and ligated into cut pK18*mobsacB*. Sequenced clones were conjugated into Rba. sphaeroides from E. coli S17-1, and transconjugants in which the clone had integrated into the genome by homologous recombination were selected on M22+ medium supplemented with kanamycin. Transconjugants that had undergone a second recombination event were then selected on M22+ supplemented with 10% (wt/vol) sucrose, lacking kanamycin. Sucrose-resistant kanamycin-sensitive colonies had excised the allelic exchange vector through the second recombination event (34). The deletion of the desired gene was confirmed by colony PCR using relevant CheckF and CheckR primers.

### Whole-cell absorption spectroscopy.

Cell pellets were resuspended in 60% sucrose to reduce light scattering, and absorption between 350 to 850 nm was recorded on a Cary 60 UV-Vis spectrophotometer.

### Extraction of pigments.

Pigments were extracted twice from cell pellets after washing in 20 mM HEPES (pH 7.2) by adding an excess of 0.2% (vol/vol) ammonia in methanol, bead beating for 30 s, and incubating on ice for 20 min (35). The extracts were then dried in a vacuum concentrator at 30°C and reconstituted in a small volume of the same solvent. The extracts were clarified by centrifugation (15,000 × *g* for 5 min at 4°C), and the supernatants were immediately analyzed on an Agilent 1200 high-performance liquid chromatography (HPLC) system.

### Preparation of Zn-BChl *a*.

Zn-BChl *a* was prepared from Mg-BChl *a* extracted from WT Rba. sphaeroides using a method modified from one previously described (36). Briefly, 1 volume of extracted Mg-BChl *a* in methanol was mixed with an excess of anhydrous zinc acetate, 50 mM sodium ascorbate, and 6 volumes of glacial acetic acid. The mixture was incubated in boiling water for 2 h in an uncapped 1.5-ml Eppendorf tube. The remaining supernatant was transferred to a new tube, clarified by centrifugation (15,000 × *g* for 5 min at 4°C), and then dried in a vacuum concentrator at 30°C. The dried pigments were reconstituted in 0.2% (vol/vol) ammonia in methanol and clarified by centrifugation as above before analysis by HPLC.

### Analysis of pigments by HPLC.

BChl *a* species were separated on a Fortis UniverSil C18 reverse-phase column (5-μm particle size, 150 mm by 4.6 mm) using a method modified from that of van Heukelem et al. (37). Solvents A and B were 80:20 (vol/vol) methanol–500 mM ammonium acetate and 80:20 (vol/vol) methanol-acetone, respectively. Pigments were eluted at 1 ml/min at 40°C on a linear gradient of 92 to 93% solvent B over 10 min, increasing to 100% to wash the column. Elution of BChl *a* species was monitored by checking absorbance at 770 nm.

### RNA isolation and quantitative real time-PCR (qRT-PCR).

Rba. sphaeroides cultures were grown in 40 ml of medium in 250-ml Erlenmeyer flasks, shaken at 150 rpm, which represents intermediate oxygenation conditions suitable for BChl *a* production. Total RNA was isolated at mid-exponential growth phase using the RNeasy Protect Bacteria minikit (Qiagen). The cell disruption was performed by treatment with 10 mg/ml lysozyme for 30 min at room temperature with constant shaking. The isolated RNA was treated with the Turbo DNA-*free* kit (Ambion) to eliminate genomic DNA contamination. One microgram of RNA was used for reverse transcription using the SensiFAST cDNA synthesis kit (Bioline) according to the manufacturer's instructions. No-RT controls were included for RNA samples by omitting the reverse transcriptase in the reaction mixture.

Gene expression levels were analyzed by qRT-PCR using the SensiFAST SYBR Lo-ROX kit (Bioline) with a Stratagene Mx3005P system (Agilent). Primers RT0294F/RT0294R were used to detect rsp_0294 transcripts, and RTrpoZF/RTrpoZR were used to detect *rpoZ* transcripts, which served as an internal reference (38). The qRT-PCRs were set up in a 20-μl volume containing 10 μl of 2× SensiFAST SYBR Lo-ROX mix, 0.4 μM primers, and 6.25 ng cDNA template. The primer efficiency was determined using 10-fold serial dilutions of genomic DNA from Rba. sphaeroides. No-RT controls and no-template controls were also included. The qRT-PCR conditions were as follows: 3 min at 95°C and 40 cycles of 5 s at 95°C and 30 s at 60°C, followed by melting curve analysis. The relative expression ratios were calculated and statistically analyzed using the REST software (39) based on the Pfaffl method (40).

## RESULTS

### Deletion of *bchE* does not reveal a functional AcsF in Rba. sphaeroides.

**FIG 3:**
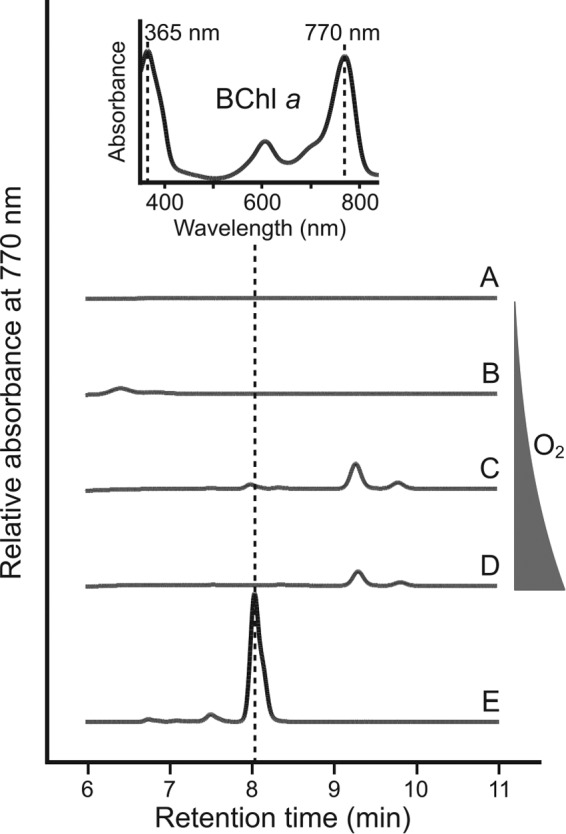
HPLC elution profiles of pigments extracted from the Δ*bchE* mutant grown under differing aeration conditions. Pigments were extracted from the Δ*bchE* strain grown at increasing aeration (A to D) as described in Materials and Methods. Pure BChl *a* (E) was used as a standard. Retention times and absorption spectra of peaks are used to identify BChl *a* (inset).

Rbv. gelatinosus is able to synthesize BChl *a* under conditions ranging from oxic to anoxic by employing O_2_-dependent and O_2_-independent cyclase enzymes, respectively (11, 12). In order to determine whether a similar situation exists in Rba. sphaeroides, in which BchE dominates but a putative *acsF* gene (rsp_0294) exists, a strain lacking *bchE* was constructed (see Fig. S1 in the supplemental material). The ability of the Δ*bchE* mutant to produce BChl *a* via AcsF under a range of oxygen tensions (see Materials and Methods) was tested by HPLC analysis of pigments extracted from cultures standardized by cell number (Fig. 3). BChl *a* production by the Δ*bchE* strain was not detected under any of the conditions tested (Fig. 3, profiles A to D), while the WT accumulates BChl *a* as expected (Fig. 3, profile E). These data suggest that a functional AcsF is not found in Rba. sphaeroides.

### Effect of the deletion of *ccoP* in Rba. sphaeroides.

**FIG 4:**
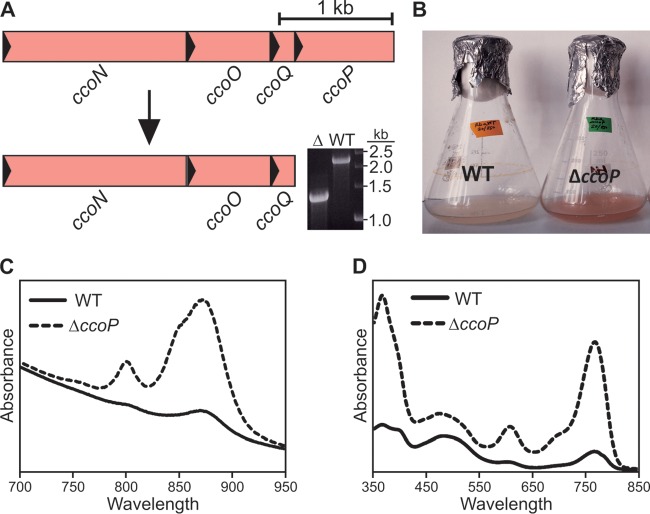
Construction and phenotype of the Δ*ccoP* strain. (A) Diagram depicting the deletion of *ccoP* and confirmation by colony PCR (inset). (B) Illustration of differential pigmentation of WT and Δ*ccoP* cultures grown at high aeration. (C and D) Whole-cell absorption spectra (C) and absorption spectra of pigments (D) extracted from WT and Δ*ccoP* strains, standardized by cell number.

The subunits of the *cbb*_3_-type terminal respiratory oxidase are encoded by the genes found in the *ccoNOQP* operon, and disruption of this stretch of open reading frames (ORFs) results in the assembly of the photosynthetic architecture under oxic conditions (22, 23). The *ccoP* gene, which encodes a membrane-bound diheme *c*-type cytochrome subunit, was deleted in the WT in order to replicate these conditions (Fig. 4A). When grown under oxic conditions achieved via high aeration, the Δ*ccoP* strain was visibly pigmented compared to the WT (Fig. 4B). Whole-cell absorption spectra of these strains standardized by cell number demonstrated that photosynthetic apparatus assembly was derepressed upon deletion of *ccoP* (Fig. 4C). Pigments extracted from these standardized samples also show that removal of *ccoP* results in greatly increased accumulation of BChl *a* in this strain relative to the WT (Fig. 4D). This background was considered to be ideal for testing the activity of RSP_0294.

### RSP_0294 activity can be detected in a strain lacking *ccoP*.

**FIG 5:**
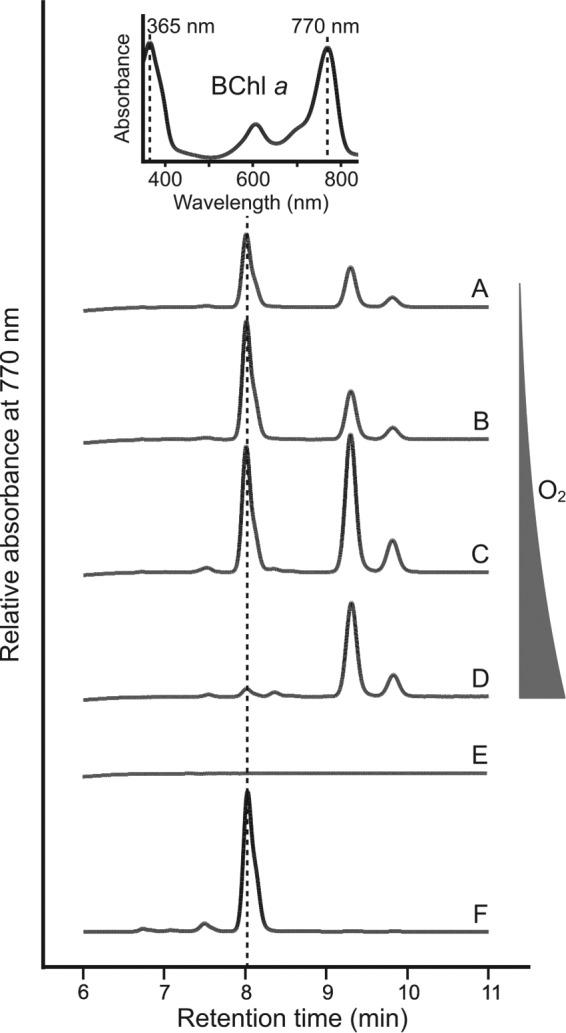
HPLC elution profiles of pigments extracted from strains lacking *ccoP* grown under differing aeration. Pigments were extracted from Δ*bchE* Δ*ccoP* strains at increasing aeration (A to D) as described in Materials and Methods. Trace (E) represents a typical elution profile from extracts of Δ*bchE* Δ*ccoP* Δrsp_0294 cells cultured under all tested conditions. Pure BChl *a* (F) was used as a standard. Retention times and absorption spectra of peaks are used to identify BChl *a* (inset).

In order to determine if the derepression of the BChl biosynthesis pathway in the Δ*ccoP* strain could reveal if RSP_0294 is an active AcsF component of the oxygen-dependent cyclase, this mutation was combined with Δ*bchE*. The resulting Δ*bchE* Δ*ccoP* strain was grown under the range of oxygen tensions described earlier for the Δ*bchE* strain, and the pigments extracted from these strains were analyzed by HPLC. Peaks corresponding to BChl *a* were present in the traces from all of the samples (Fig. 5, profiles A to D), although the BChl *a* extracted from the Δ*bchE* Δ*ccoP* strain grown at the highest oxygen tensions approached the detection limit of the instrument (Fig. 5, profile D). To confirm that the cyclization reaction in this strain was dependent on the presence of RSP_0294, this ORF was deleted in the Δ*bchE* Δ*ccoP* strain (see Fig. S2 in the supplemental material). The resulting strain, Δ*bchE* Δ*ccoP* Δrsp_0294 mutant, was again cultured under the previously described oxygen tensions. Extracts from each culture contained no detectable BChl *a* (Fig. 5, profile E). These data confirm that oxygen-dependent cyclase activity in Rba. sphaeroides is reliant on the presence of RSP_0294 and that this protein is the active AcsF component of the enzyme. We therefore propose that rsp_0294 be reassigned as *acsF*.

### Deletion of *ccoP* slightly increases the expression level of *acsF*.

The expression levels of *acsF* in WT, Δ*bchE*, and Δ*bchE* Δ*ccoP* strains of Rba. sphaeroides were analyzed by qRT-PCR. Total RNA was isolated from cultures grown to provide the optimum conditions for BChl *a* production based on the HPLC results. Each qRT-PCR was performed in triplicate. The housekeeping gene *rpoZ*, encoding the ω-subunit of RNA polymerase, was included as an internal reference (38). The primer efficiency was deduced from a standard curve generated by using genomic DNA as a PCR template in a series of 10-fold dilutions. These efficiencies were 99.09% for *acsF* and 97.35% for *rpoZ*. The primer specificity and the absence of primer dimers were confirmed by melting curve analysis. By giving the WT a value of 1, the relative expression ratio of *acsF* was calculated using the threshold cycle deviation between a mutant strain and the WT, with primer efficiency correction and normalization to the internal reference gene *rpoZ*. The pairwise fixed reallocation randomization test was performed to test whether there was a significant difference between the described mutants and the WT. As shown in Table 2, the expression level of *acsF* in the Δ*bchE* mutant was not significantly different from the level of the WT (*P* = 0.1). However, *acsF* expression was increased by a factor of 2.293 in the Δ*bchE* Δ*ccoP* strain, a significant increase (*P* < 0.05) compared to WT. Thus, deletion of *ccoP* results in increased expression of *acsF*; this result agrees with data indicating that the *cbb*_3_-type cytochrome *c* oxidase, encoded by the *ccoNOQP* operon, can generate an inhibitory signal to repress photosynthesis gene expression in Rba. sphaeroides (23).

**TABLE 2:** Expression levels of *acsF* in described strains as determined by qRT-PCR^*a*^

Rba. sphaeroides strain	Expression level	95% confidence interval	P(H1)
WT	1	NA	NA
Δ*bchE* mutant	1.202	1.004–1.470	0.1
Δ*bchE* Δ*ccoP* double mutant	2.293	1.839–3.247	0.017

a P(H1) represents the probability of the alternative hypothesis that the difference between a mutant and the WT is due only to chance. NA, not applicable.

### Zn-BChl *a* accumulates in Δ*bchE* mutants of Rba. sphaeroides.

**FIG 6:**
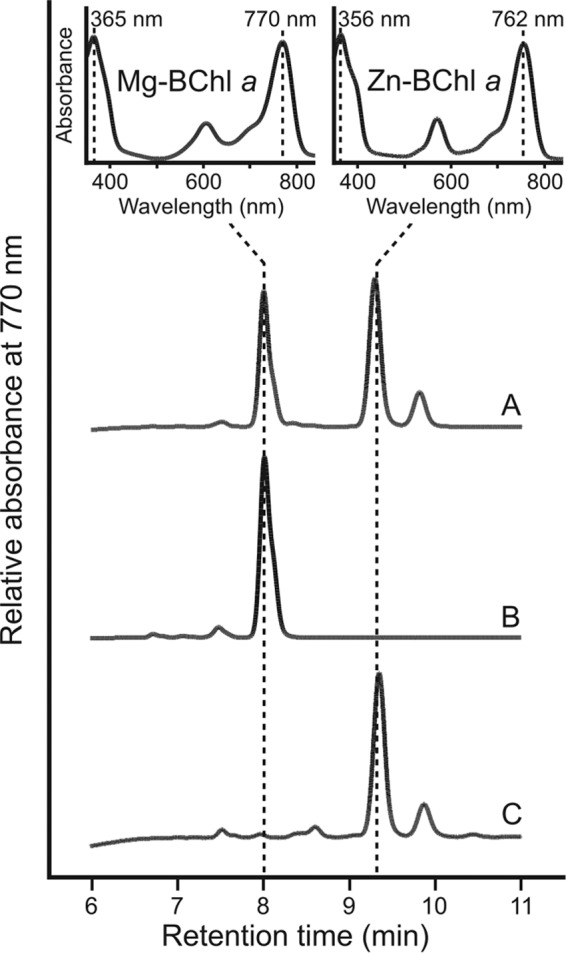
HPLC elution profiles of pigments extracted in order to assign the peak at 9.3 min. (A) Pigments extracted from Δ*bchE* Δ*ccoP* cells cultured in 40 ml of medium in a 250-ml Erlenmeyer flask. (B and C) Mg-BChl *a* extracted from WT (B) and Zn-BChl *a* (C), prepared as described in the text. Retention times and Soret/Q_y_ maxima of peaks are used to identify BChl *a* species (inset).

In both the presence and absence of *ccoP*, the Δ*bchE* mutant accumulates a pigment that can be detected by absorbance at 770 nm and has a retention time longer than that of BChl *a* (see peak at 9.3 min in Fig. 3 and 5), properties indicative of a bacteriochlorin pigment carrying a hydrophobic alcohol moiety, the addition of which is the last step in mature photopigment production. It has been demonstrated that an Rba. sphaeroides Tn*5* mutant in a gene encoding a subunit of magnesium chelatase (*bchD*), the enzyme catalyzing the first committed step in BChl biosynthesis, is able to assemble photosynthetic apparatus containing Zn-BChl *a* (41, 42), indicating that the BChl biosynthetic enzymes demonstrate plasticity with regard to the divalent metal within the pigment macrocycle. To determine whether the pigment accumulated in the Δ*bchE* strains was Zn-BChl *a*, this pigment was prepared from Mg-containing BChl *a* extracted from WT Rba. sphaeroides via an acid reflux method described previously by Hartwich et al. (36) (see Materials and Methods). The retention time and absorption spectrum of the prepared Zn-BChl *a* were identical to those of the 9.3 min peak in the pigments extracted from the highly aerated Δ*bchE* Δ*ccoP* strain (Fig. 6). The longer retention time, as well as blue shifts in both the Soret and Q_y_ absorption bands of Zn-BChl *a*, compared to Mg-BChl *a*, are in agreement with published data (42).

## DISCUSSION

Here we demonstrate that the model purple phototrophic bacterium Rba. sphaeroides is able to utilize both oxygen-dependent and oxygen-independent forms of the cyclase enzyme for the biosynthesis of BChl *a*. The activity of the oxygen-dependent form was revealed after the expression of genes involved in photosynthetic processes was derepressed under high aeration by the inactivation of the *cbb*_3_ terminal respiratory oxidase, when we were able to detect Mg-BChl *a* in a strain lacking the dominant, oxygen-sensitive enzyme. This derepression led to a >2-fold increase in the level of *acsF* transcript. The inactivation of the *cbb*_3_ oxidase was initially the difference between undetectable and apparent Mg-BChl *a* accumulation, although the increase in *acsF* expression was relatively modest. Therefore, it may be that the large increase in the amount of Mg-BChl *a* in particular, but also Zn-BChl *a*, formed in the Δ*bchE* Δ*ccoP* mutant was due not only to this increased expression of *acsF* but also to the increase in available substrate for this enzyme; in WT cells, O_2_ is both the terminal electron acceptor for the *cbb*_3_ oxidase, which it binds with high affinity, and a substrate for the oxidative cyclase; thus, the competition for O_2_ in a strain lacking *ccoP* is greatly reduced.

In addition to *cbb*_3_, Rba. sphaeroides also employs a lower-affinity *aa*_3_-type terminal respiratory oxidase (26). The genes encoding the subunits of this cytochrome could also have been viable targets for disruption in order to increase the cellular level of O_2_. However, proteomic work published by our laboratory has indicated that the majority of the enzymes involved in tetrapyrrole biosynthesis are found concentrated in the precursor of the mature, photosynthetic apparatus-containing intracytoplasmic membrane (43). The precursor membrane, known as UPB (upper pigmented band, migrating slower than intracytoplasmic membranes in rate zone sedimentation), was also found to contain the majority of the *cbb*_3_ oxidase, unlike other proteins involved in photosynthetic energy transduction, which were all more abundant in the intracytoplasmic membrane. These data suggested proximity between the *cbb*_3_ oxidase and enzymes involved in BChl biosynthesis; thus, we chose this cytochrome as our target for disruption.

Deletion of *ccoP* led to the detectable accumulation of Mg-BChl *a* in the Δ*bchE* mutant, although further analysis revealed the presence of Zn-BChl *a* in the Δ*bchE* strain in both the presence and absence of *ccoP*. The occurrence of BChls containing zinc rather than magnesium has been previously documented. It has been discovered that when cultured heterotrophically to late stationary phase in acidic medium, the unicellular alga Chlorella kessleri accumulates Zn-Chl *a* (44). Additionally, the acidophilic aerobic anoxygenic phototroph Acidiphilium rubrum assembles functional light-harvesting apparatus solely with Zn-BChl *a* (45). It was subsequently shown that the magnesium chelatase enzyme of this organism catalyzed insertion of Mg^2+^ into the pigment macrocycle (46), and thus it was proposed that the insertion of Zn follows dechelation of Mg at a later stage in the biosynthesis of the photopigment. Recently, the first phototroph identified from the phylum Acidobacteria, Chloracidobacterium thermophilum, was discovered to contain both Mg- and Zn-BChls *a* in its homodimeric type I photosynthetic reaction center, although the exact role of each pigment is not currently known (47). It was hypothesized that, in the absence of an active magnesium chelatase enzyme, the accumulation of Zn-BChl *a* in the *bchD* mutant of Rba. sphaeroides was due to insertion of Zn^2+^ into the macrocycle of protoporphyrin IX, catalyzed by ferrochelatase (42). The *in vivo* role of this enzyme is the insertion of Fe^2+^ into protoporphyrin IX during the biosynthesis of hemes, but it has been shown to chelate Zn^2+^ *in vitro* (48, 49). However, the strains described in this study contain a functional magnesium chelatase enzyme, and accumulation of Mg-BChl *a* in the Δ*bchE* Δ*ccoP* strain suggests that, as in the cases of Chlorella kessleri and Acidiphilium rubrum, zinc insertion may occur after dechelation of magnesium. We propose that the high O_2_ tension in the Δ*bchE* strains, containing or lacking *ccoP*, coupled with the low level of Mg- or Zn-chelated BChl *a* formed via the AcsF route, prevents assembly of the photosynthetic apparatus. It may be that the unbound Mg-BChl *a* is susceptible to dechelation, either by a spontaneous reaction or catalyzed by an as-yet-unidentified dechelatase enzyme, while bound Mg-BChl *a* in the photosynthetic apparatus may be effectively shielded from this process.

The presence of Zn-BChl *a* in the Δ*bchE* mutant indicates that AcsF-catalyzed formation of the Mg-chelated pigment occurs under oxic conditions, but replacement of the central metal of the unbound Mg-BChl occurs with high efficiency. Although the overall contribution to BChl *a* biosynthesis is small, the role of AcsF in Rba. sphaeroides may be to ensure a modest level of mature pigment in cells switching from aerobic respiration to phototrophy. BChl biosynthesis is likely initiated at the indented regions of the cytoplasmic membrane identified as sites for preferential synthesis of BChl and photosystem apoproteins (21, 50–52). AcsF might ease the transition from oxic growth, by providing BChl for the earliest stage of photosystem assembly, which mainly involves the synthesis of the reaction center-light harvesting 1-PufX complex (53, 54).

We have demonstrated that Rba. sphaeroides, like Rbv. gelatinosus, is able to use both oxygen-dependent and oxygen-independent cyclases for BChl biosynthesis. Many other purple phototrophs contain genes assigned to both enzymes, and it may be that the ability to utilize both forms of the enzyme for pigment production, or to switch between them according to the balance of oxic versus anoxic conditions, is conserved in these strains. Outside the purple bacteria, organisms containing orthologs of both *bchE* and *acsF* have been reported. The green filamentous anoxygenic phototroph Chloroflexus aurantiacus primarily relies on BchE for the production of BChls, yet AcsF rather than BchE can been detected in the specialized chlorosome antenna under anoxic conditions, and unlike *bchE*, expression of *acsF* does not change with O_2_ tension (16). These observations led the authors to hypothesize alternative functions for AcsF in C. aurantiacus; the diiron protein may have evolved to play a role in electron transfer or iron transport under anoxic conditions. Conversely, the cyanobacterium Synechocystis sp. strain PCC 6803 relies on different AcsF proteins for Chl *a* biosynthesis under oxic and micro-oxic conditions but contains three orthologs of *bchE*, none of which appears to play a role in pigment production under any oxygen tension (15). Recently, cyanobacterial *bchE* orthologs from two strains of Cyanothece were shown to be able to restore BChl *a* biosynthesis in a *bchE* mutant of R. capsulatus, demonstrating activity of oxygen-independent ChlE proteins from oxygenic phototrophs for the first time (55). Boldareva-Nuianzina et al. propose that *acsF* was adopted by the *bchE*-containing proteobacteria via horizontal gene transfer from cyanobacteria, in which this gene evolved (17). They suggest that acquisition after the Great Oxygenation Event in the early Proterozoic era, when the surface of the oceans became mildly oxic while deep waters remained anoxic, allowed the early purple phototrophs to adapt to these new conditions in water supporting both oxygenic and anoxygenic photosynthesis (56).

## Supplementary Material

Supplemental material
