# Supplementary material for: Absence of the cbb3 Terminal Oxidase Reveals an Active Oxygen-Dependent Cyclase Involved in Bacteriochlorophyll Biosynthesis in Rhodobacter sphaeroides
Source: J Bacteriol. 2016 Jul 13;198(15):2056–63. doi: 10.1128/JB.00121-16 (PMC4944227; doi:10.1128/JB.00121-16)
Supplement: Supplemental material [file supp_198_15_2056__index.html]

Supplemental material 

# Absence of the *cbb*3 Terminal Oxidase Reveals an Active Oxygen-Dependent Cyclase Involved in Bacteriochlorophyll Biosynthesis in Rhodobacter sphaeroides

## Supplemental material

- Supplemental file 1 -

  Table S1 (Primers) and Fig. S1 (Construction of Δ*bchE*) and S2 (Construction of Δrsp\_0294)

  PDF, 434K
