# Supplementary material for: Absence of the cbb3 Terminal Oxidase Reveals an Active Oxygen-Dependent Cyclase Involved in Bacteriochlorophyll Biosynthesis in Rhodobacter sphaeroides
Source: J Bacteriol. 2016 Jul 13;198(15):2056–63. doi: 10.1128/JB.00121-16 (PMC4944227; doi:10.1128/JB.00121-16)
Supplement: Supplemental material [file JB.00121-16_zjb999094116so1.pdf]

## SUPPLEMENTARY MATERIAL

| Primer | Sequence (5'-3') | Restriction site |
|---|---|---|
| bchEUpF | CTGCCCGGGCGACGGGCGTGATCGACGAGCCC | SmaI |
| bchEUpR | GGAATTCCATATGTGGACTCCCGCTGTGTCCATTTC | NdeI |
| bchEDownF | GGAATTCCATATGACCGCGCATGACCAGCGG | NdeI |
| bchEDownR | GCAAGCTTGGAATGTTTGGCGATGGCCGTGG | HindIII |
| bchECheckF | GCAGATTGCCGCAGAGATCTCG | |
| bchECheckR | GGTTCCGGGCAATCTCGAATGAC | |
| ccoPUpF | CCGGAATTCGTTCCTCTCGCACACCGTGATC | BamHI |
| ccoPUpR | GGATTACTCACTCATTTCCTCGCCTCCTCGG | |
| ccoPDownF | GAAATGAGTGAGTAATCCAAGGAGCTGAAGCGG | |
| ccoPDownR | CCGCAAGCTTCAGATCGACGAGGATCGCCTG | HindIII |
| ccoPCheckF | CTACGTCTGTCACAGCCAGATGATC | |
| ccoPCheckR | GCTCGACGAGGATGAAGAGATCG | |
| 0294UpF | CGCGGATCCCCTTCGAGCGGATGCTGTCC | EcoRI |
| 0294UpR | CCGGTGATCGTCAGAAGTCACATATGGTCACCTGCTCGGAGAAGGAG | |
| 0294DownF | CTCCTTCTCCGAGCAGGTGACCATATGTGACTTCTGACGATCACCGG | |
| 0294DownR | CCCAAGCTTCCCGTGATGACGCCCGACAGG | HindIII |
| 0294CheckF | CCGAGCTCCAGGCATTCGGACC | |
| 0294CheckR | GCCCGCAGGAATCGCTCGG | |
| RT0294F | ATCGCTTCCACCCGATCTTC | |
| RT0294R | CGGATCGGTCTTCATCAGCA | |
| RTrpoZF | GACGGTTGAAGACTGCGTTG | |
| RTrpoZR | GTTCTTGTCATTGTCGCGGT | |

**Table S1. List of primers used in this study**

Restriction sites used for cloning are underlined in the sequence.

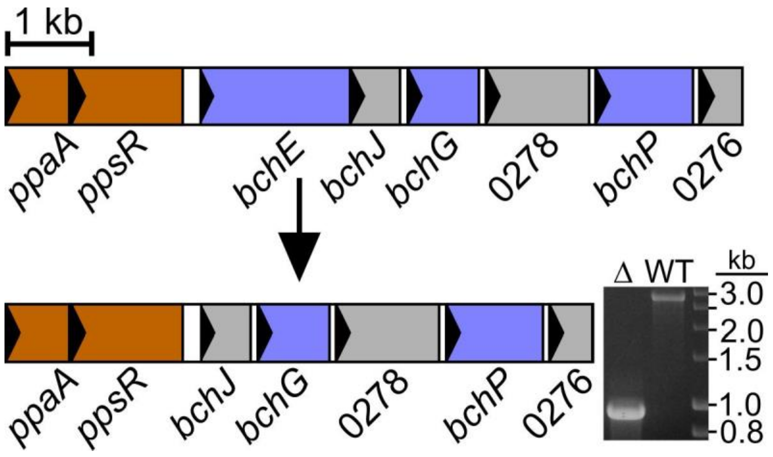


**Fig. S1. Construction of Δ*bchE***

Diagram depicting the deletion of *bchE* and confirmation by colony PCR (inset). Genes encoding BChl biosynthesis enzymes and regulatory elements are colored in blue and amber, respectively. ORFs encoding proteins of unknown function are colored in grey. Numbered loci are prefixed with 'rsp_' (e.g. 0278 = rsp_0278).

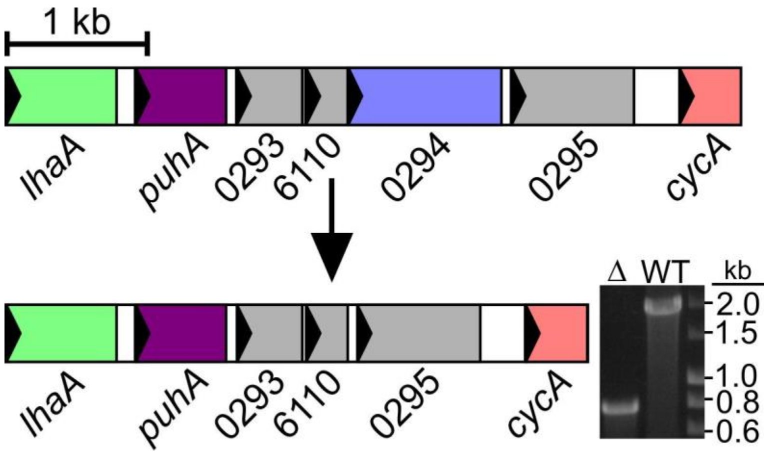


**Fig. S2. Construction of Δrsp_0294**

Diagram depicting the deletion of rsp_0294 and confirmation by colony PCR (inset). Genes encoding BChl biosynthesis enzymes, assembly factors, reaction center subunits and cytochromes are blue, green, purple and pink, respectively. ORFs encoding proteins of unknown function are colored in grey. Numbered loci are prefixed with ‘rsp_’ (e.g. 0293 = rsp_0293).
